# A Novel Immunoassay for Malondialdehyde-Conjugated Low-Density Lipoprotein Measures Dynamic Changes in the Blood of Patients Undergoing Coronary Artery Bypass Graft Surgery

**DOI:** 10.3390/antiox10081298

**Published:** 2021-08-17

**Authors:** Samata S. Pandey, Adam Hartley, Mikhail Caga-Anan, Tareq Ammari, Ameer Hamid Ahmed Khan, Bao Anh Vu Nguyen, Chiari Kojima, Jon Anderson, Steven Lynham, Michael Johns, Dorian O. Haskard, Ramzi Y. Khamis

**Affiliations:** 1 Vascular Sciences Section, National Heart and Lung Institute, Imperial Centre for Translational and Experimental Medicine, Imperial College London, London W12 0NN, UK; samata.pandey12@imperial.ac.uk (S.S.P.); adam.hartley12@imperial.ac.uk (A.H.); m-caga.anan@imperial.ac.uk (M.C.-A.); Tareq.ammari@gmail.com (T.A.); ameerhamidahmedkhan@gmail.com (A.H.A.K.); bao.nguyen@doctors.net.uk (B.A.V.N.); c.kojima@imperial.ac.uk (C.K.); m.johns@imperial.ac.uk (M.J.); d.haskard@imperial.ac.uk (D.O.H.); 2 Department of Cardiothoracic Surgery, Hammersmith Hospital, Imperial College Healthcare NHS Trust, London W12 0HS, UK; jonr.anderson@nhs.net; 3 Centre of Excellence for Mass Spectrometry, Proteomics Facility, Denmark Hill Campus, Kings College London, London SE5 9NU, UK; steven.lynham@kcl.ac.uk

**Keywords:** atherosclerosis, low-density lipoprotein (LDL), oxidized LDL, malondialdehyde, anti-oxidized LDL antibodies, antibodies, assay, inflammation

## Abstract

Oxidized low-density lipoproteins play an important role in tissue pathology. In this study, we report a sensitive novel enzyme-linked immunosorbent assay for the detection of malondialdehyde-modified low-density lipoprotein (MDA-LDL), a key component of oxidized LDL. The assay is capable of measuring a variable presence of MDA-LDL within human plasma and serum. We demonstrate the robust nature of the assay on samples stored for over 20 months, as well as high inter-operator reproducibility (r = 0.74, *p* < 0.0001). The assay was capable of detecting dynamic changes in patient blood samples after coronary artery bypass graft surgery, indicating synthesis or release of MDA-LDL with the oxidative stress of surgery, followed by homeostatic clearance. This robust, sensitive and specific assay for circulating MDA-LDL will serve as a valuable translational tool for the improved detection of oxidative forms of LDL in response to a range of physiological or pathological stimuli, with potential clinical applicability.

## 1. Introduction

Oxidation-specific epitopes (OSE) on lipoproteins are an important class of danger-associated molecular patterns that promote cellular dysfunction and death. Moreover, such changes render LDL antigenic, leading to uptake by macrophage scavenger receptors, and resulting in apoptosis, foam cell formation, atherosclerotic lipid core development and inflammatory cytokine production [1,2,3]. In addition, oxidized LDL (oxLDL) accumulation has been identified in post-mortem studies as a key feature of atherosclerotic plaques prone to rupture [4].

The central role of oxLDL in atherosclerosis and other tissue pathology has led to the development of monoclonal antibodies (mAb) binding various OSEs and their use in assays that measure circulating oxLDL levels. These assays have gained greater significance now that multiple studies have demonstrated that higher plasma levels of oxLDL are associated with incident cardiovascular disease (CVD) [5]. Indeed, a meta-analysis has demonstrated that higher plasma oxLDL levels are related to atherosclerotic CVD, with a significant effect size of 1.79 (95% CI, 1.56–2.05) [6]. Thus, oxLDL in general may be a clinically meaningful biomarker of cardiovascular risk.

One assay to detect oxLDL utilizes mAb 4E6, which recognizes a conformational epitope on Apolipoprotein-B (ApoB)-100 resulting from the substitution of 60 lysine residues to aldehydes [7,8,9]. Other assays have used mAb DLH3 and E06, which bind oxidized phospholipids through their recognition of oxidized phosphatidylcholine and the hydrophilic moiety of phosphatidylcholine, phosphorylcholine, respectively [10,11]. Given the heterogenous nature of oxLDL and disparate assay development, assays are not easily comparable, and this is further complicated by different units of measurement in each assay.

We have previously described the isolation of a spontaneously arising murine IgG3 κ anti-oxLDL mAb, named LO1. It recognizes malondialdehyde (MDA)-conjugated LDL, and only minimally reacts with “native” LDL or to an alternative MDA-conjugated carrier (albumin) [12,13]. Through immunohistochemical staining, we have shown that LO1 reacts with antigen in tissue sections of mouse and human atherosclerosis, with binding prevented by free MDA-LDL. We have performed further validation of antibody specificity using in vivo imaging studies. LO1 has been conjugated with a near-infrared fluorophore and injected in mice and rabbits in vivo, with near-infrared fluoroscopic imaging performed using whole-body or intra-arterial catheter fluorescence molecular tomography respectively [13]. Here, we extend the application of LO1 by showing that it can be used in a reproducible sandwich ELISA to detect MDA-LDL in plasma. Furthermore, we have used the novel assay on blood samples from a patient population undergoing coronary artery bypass grafting (CABG) to show that it has the potential for use as a clinical biomarker.

## 2. Materials and Methods

### 2.1. Generation of MDA-LDL

Preparation of MDA-LDL was carried out as described by Palinski et al [14]. Briefly, MDA was produced through rapid acid hydrolysis of 1,1,3,3-tetramethoxypropane (Sigma-Aldrich, Poole, UK) for 10 min at 37 °C using 4M of HCl. The pH was adjusted to pH7.4 using 1.5M NaOH creating a stock solution of 0.5M MDA. Human plasma LDL (Merck Millipore, Darmstadt, Germany) was incubated with MDA for three hours at 37 °C at a ratio of 100 μL of 0.5 MDA to 1 mg of LDL. ZebaSpin Desalting columns (7K MWCO; ThermoScientific, Loughborough, UK) were used to buffer exchange into PBS. Concentration of MDA-LDL was probed with the use of Pierce BCA Protein Assay Kit (ThermoFisher Scientific, Loughborough, UK) according to manufacturer’s instructions. 0.01% EDTA was added to prevent further oxidation.

### 2.2. Generation of LO1

LO1 was purified from hybridoma culture supernatants by affinity chromatography on protein G sepharose as previously described [12]. Non-specific IgG3 κ was used as an isotype control (Sigma Aldrich, Poole, UK).

### 2.3. Sandwich Enzyme-Linked Immunosorbent Assay (ELISA) to Detect MDA-LDL

Nunc Maxisorp plates (ThermoFisher Scientific, Waltham, MA, USA) were coated with 100 μL/well LO1 capture antibody in Dulbecco’s Phosphate Buffered Saline (DPBS, Gibco) at room temperature (RT) overnight. Plates were washed with Wellwash™ Microplate Washer (Thermo), using 3 cycles of 300 µL/well 0.05% Tween in DPBS per wash, after each of the incubation steps to remove non-adherent material. Plates were blocked with 300 µL/well of 2% Bovine Serum Albumin (BSA) (Sigma Aldrich, Poole, UK) for 1 h at RT. Reagent diluent (DPBS with 0.5% BSA and 0.05% tween) was used to produce the required concentrations of reagents. Antibody and plasma layers were incubated for 1 h whereas streptavidin-HRP was incubated for 20 min. 3,3′,5,5′-tetramethylbenzidine (TMB) (Sigma-Aldrich, Poole, UK) was used to develop the ELISA and stopped with equal volume of sulphuric acid (0.5 M H_2_SO_4_). The optical density (OD) was measured at 450 nm using a synergy HT multi-mode microplate reader (Southern Biotech, Birmingham, AL, USA). On testing patient plasma samples, reference plasma from a healthy volunteer was used to correct against in a 2-in-1 dilution series. Values were corrected to background measured using 2% BSA as a negative control.

### 2.4. Mercodia oxLDL Assay

The Mercodia assay for oxLDL (Mercodia, Uppsala, Sweden) was used according to the manufacturer’s instructions.

### 2.5. Direct ELISAs

Briefly, as previously described [12], Nunc Maxisorp plates (ThermoFisher Scientific, Waltham, MA, USA) were coated with LO1 overnight at room temperature (RT) at 10 μg/mL. Subsequently, the plates were blocked in 2% BSA and then incubated with MDA-LDL for 1 h followed by a polyclonal anti-Apolipoprotein B-HRP (Abcam, Cambridge, UK) and developed with TMB (SigmaAldrich, Poole, UK). Plates were washed 3-times in 0.05% DBPS-Tween20 between steps.

### 2.6. ApoB ELISA

Equal optimal concentrations of plasma and anti-ApoB-HRP antibody as described above were used (i.e., 1:40 and 1:2000 respectively). Testing a 2-fold dilution series (starting at concentration of 1:500) of capture antibody polyclonal goat anti-human ApoB with synthetic MDA-LDL and plasma, the optimal concentration of capture anti-ApoB antibody was determined as 1:5000.

### 2.7. IgG and IgM Anti-MDA-LDL ELISAs

MDA-LDL at a concentration of 10 µg/mL was adhered to plates for detecting anti-MDA-LDL antibodies. The sample dilutions were 1:80 and 1:120 for total, IgG and IgM assays, respectively. The primary detection antibodies were either an unlabelled mouse anti-human IgG (Southern Biotech, Birmingham, AL, USA) or a biotinylated mouse anti-human IgM (Southern Biotech, Birmingham, AL, USA), both at dilutions of 1:2000 to measure IgG and IgM anti-MDA-LDL antibodies, respectively. The secondary detection antibody was an HRP-conjugated polyclonal rabbit anti-mouse antibody (Dako, Cambridge, UK) at a dilution of 1:2000 for the IgG anti-MDA-LDL assay, and an HRP-conjugated streptavidin at a dilution of 1:200 for IgM anti-MDA-LDL.

### 2.8. Lp (a) Assay

Human Lipoprotein A ELISA Kit (Abcam, Cambridge, UK) was used according to manufacturer’s instructions.

### 2.9. Freeze-Thaw Stability

The plasma samples were pooled for each donor. The original samples were thawed at room temperature (RT) and re-frozen at −80 °C after one hour, with the freeze-thaw cycle repeated at four 24-h intervals and aliquots taken during each cycle to yield five freeze-thaw samples. All samples were then finally thawed and assayed at the same time.

### 2.10. Immunoprecipitation

Samples were diluted in assay buffer (0.5% BSA + 0.05% Tween20) and incubated with LO1 (2 μg) at 4 °C overnight, followed by the addition of Pan Mouse IgG Dynabeads (Invitrogen, ThermoFisher Scientific, Waltham, MA, USA) and then incubated at 4 °C for 1 h. The beads were separated using magnetic separation and washed 5 times in PBS with 0.05% Tween20. Bound proteins were then eluted by heating for 2 min.

### 2.11. Gel Electrophoresis

Native LDL in lithium dodecyl sulphate (LDS) (NuPAGE^®^ LDS Sample Buffer (4X), Life) was heated for 2-min at 90 °C and stored at −20 °C. Proteins were separated by gel electrophoresis in 10% SDS, using precast 3–8% Tris-Acetate gels and Tris-Acetate buffer (NuPAGE Tris-Acetate SDS running buffer; Life Technologies, ThermoFisher Scientific, Waltham, MA, USA). Gels were run at 25 Volts for 2.5 h, prior to rinsing ×3 with deionized water and stained with Coomassie Brilliant Blue (SimplyBlue^TM^ SafeStain, ThermoFisherScientific, Loughborough, UK) for 1-h on a shaker. Gels were then de-stained overnight with deionized water and imaged on an optical gel reader.

### 2.12. Protein Identification Using Liquid Chromatography Mass Spectrometry/Mass Spectrometry (LC-MS/MS)

Protein identification was performed by the Centre of Excellence for Mass Spectrometry, Kings College London. Briefly, proteins were excised from the gel and reduced, carbmidomethylated, and digested in situ overnight with dithiothreitol, iodoacetamide and trypsin, respectively. Peptides were extracted using a series of acetonitrile and aqueous washes prior to being pooled and lyophilized. Samples were resuspended in 50 mM ammonium bicarbonate prior to analysis by LC-MS/MS. Chromatographic separations were performed using an EASY NanoLC system (ThermoFisherScientific, Loughborough, UK). Peptides were resolved by reversed phase chromatography on a 75 μm C18 column using a three-step linear gradient of acetonitrile in 0.1% formic acid at a flow rate of 300 nL/min. The eluate was ionised by electrospray ionisation using an Orbitrap Velos Pro (ThermoFisherScientific, Loughborough, UK) operating under Xcalibur v2.2. The instrument was programmed to acquire in automated data-dependent switching mode, selecting precursor ions based on their intensity for sequencing by collision-induced fragmentation using a Top20 CID method. Samples were searched using an All Taxonomy search of the Uniprot database through the Mascot searching algorithm. Database generated files were uploaded into Scaffold 4 (v4.4.6) software (www.proteomesoftware.com, accessed on 19 July 2021). Statistical filtering was set to 95% stringency.

### 2.13. Population Study

Blood plasma specimens were obtained from a prospective clinical study conducted at Hammersmith Hospital, Imperial College Healthcare NHS Trust, UK, that was performed to assess inflammatory responses following CABG. Patients referred for primary elective CABG surgery were considered for enrollment in accordance with the inclusion and exclusion criteria (Supplementary Table S1). All patients arrived 24-h prior to the time of operation. Surgical preparation involved fasting from midnight followed by pre-medication and antibiotic prophylaxis on the morning of the operation with baseline blood sampling. All patients received high-flow oxygen prior to anesthetic induction and intubation. Central venous and arterial access was gained, and a second post-anaesthetic induction blood specimen was taken. Blood specimens were then taken at different time intervals following commencement of cardiopulmonary bypass. For the purposes of this study, we only tested samples at baseline and at 60, 120 and 300 min (*n* = 10). Plasma samples were all stored at −80 °C and thawed to RT prior to use in ELISA. For this serology study, we randomly chose a subpopulation of those patients undergoing conventional cardiopulmonary bypass.

Ethical approval for the study was granted by local research ethics committee prior to commencement (REC reference 08/H70708/67). All patients gave written informed consent. The United Kingdom Multi-Centre Ethics Committee also granted ethical approval.

### 2.14. Statistical Analyses

Statistical analysis was performed on GraphPad Prism 8 (La Jolla, CA, USA). Normality was tested using the D’Agostino & Pearson omnibus normality test. Optical density values were expressed as mean ± standard error of the mean (SEM). Paired parametric data was assessed using the paired *t*-test, and unpaired parametric data was assessed using the unpaired *t*-test. Non-parametric paired data was analyzed using the Wilcoxon matched-pairs signed rank test and non-parametric unpaired data were analyzed using the Mann- Whitney test. Spearman correlation coefficient was used to compare LO1 capture and 4E6 assays. A *p*-value of <0.05 was considered statistically significant.

## 3. Results

**Figure 1:**
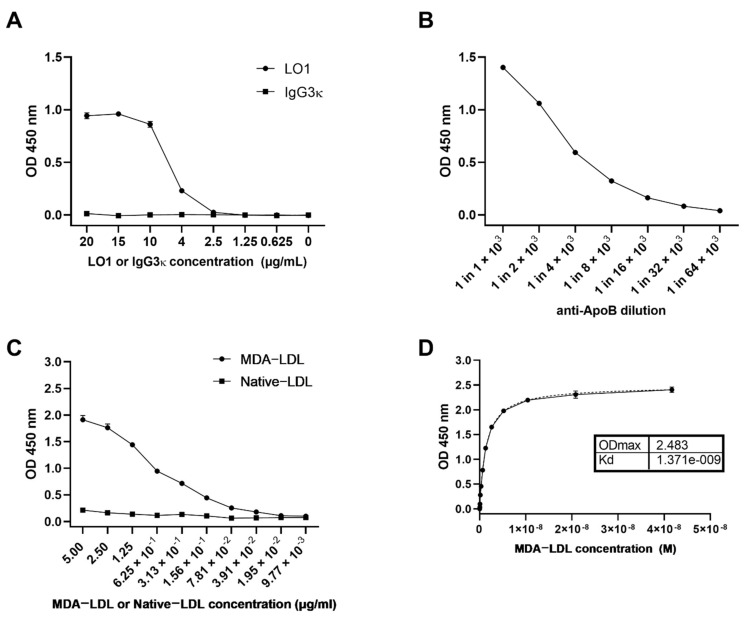
**LO1 binds MDA-LDL in fluid phase.** (**A**) LO1, but not IgG3 κ isotype control bound immobilized MDA-LDL in an ELISA; (**B**) Determination of the optimal concentration of polyclonal goat anti-ApoB-HRP detection antibody by testing a two-fold dilution series against immobilised MDA-LDL; (**C**) LO1 captured fluid phase MDA-LDL in a sandwich ELISA, but not native LDL; (**D**) Affinity and maximal optical density determination of LO1 to MDA-LDL. Values are mean ± SEM.

As previously described, the LO1 mAb reacts in vitro with immobilized MDA-LDL (Figure 1A) [12,13]. We hypothesized that LO1 could be used to detect MDA-LDL and its complexes in patient plasma samples. We therefore developed a sandwich ELISA using assay plates pre-coated with LO1 to capture fluid-phase MDA-LDL and an HRP-conjugated polyclonal anti-human ApoB antibody for detection. The anti-ApoB antibody concentration used was determined empirically within the linear range (Figure 1B). The working range for the detection of MDA-LDL in our assay is 7 × 10^−2^ to 2.5 µg ml^−1^, and the assay failed to detect native LDL (Figure 1C). The equilibrium dissociation constant (K_D_) was estimated to be 1.371 nM whilst the maximum optical density (OD_max_) was 2.483 OD units (Figure 1D).

**Figure 2:**
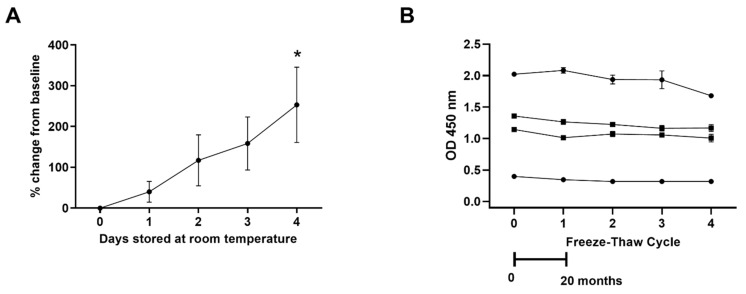
**Stability of antigen in human plasma.** (**A**) Percentage change of signal over time of plasma samples (n = 7) stored at room temperature; (**B**) Stability of MDA-LDL levels was demonstrated in four different plasma samples over four freeze-thaw cycles. Values are mean ± SEM. * = *p* <0.05 for Day 4 versus Day 0.

The reproducibility of the assay using different preparations of MDA-LDL was robust (data not shown). Increases in spontaneous oxidation levels were detected in plasma samples subjected to bench-top oxidation serially over a four-day period (Figure 2A). However, the antigen was stable in samples frozen at −80 °C and not affected by repeated freeze-thaw cycles over a 20-month period (Figure 2B).

**Figure 3:**
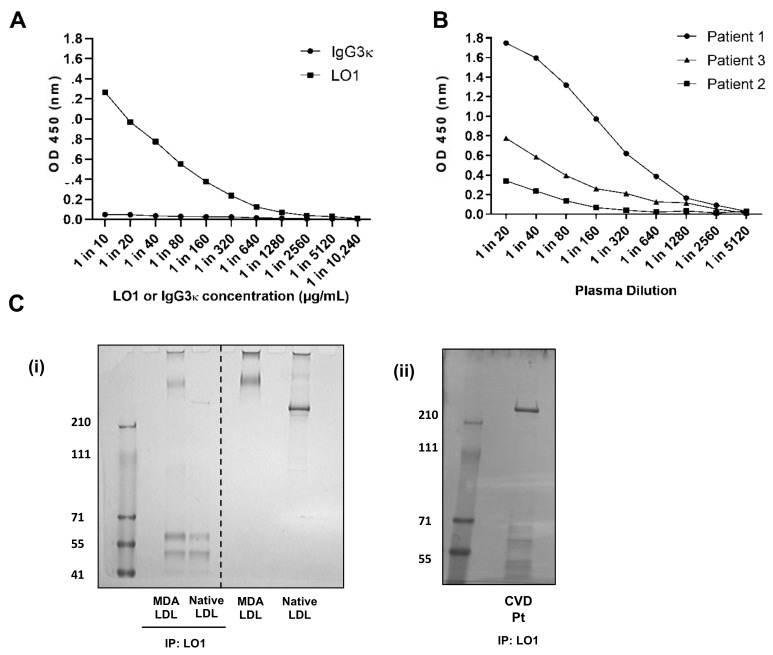
**LO1 can detect varying levels of antigen in patient samples.** (**A**) Immobilised LO1, unlike isotype control IgG3 κ, captured antigen from a plasma sample; (**B**) Immobilised LO1 captured varying levels of antigen from different patient samples; (**Ci**) Coomassie stained gel showing LO1 immunoprecipitated native LDL and in vitro-generated MDA-LDL. The top bands are likely to represent ApoB, whilst the bottom bands are likely to be degraded antibody falling off the column. The right-side of the gel shows both native LDL and MDA-LDL, with MDA-LDL showing a higher kD. (**Cii**) a second Coomassie stained gel with human plasma from a patient (Pt) with cardiovascular disease (CVD) after immunoprecipitating with LO1. The band >210kD was confirmed to be mainly ApoB-100 by mass spectrometry (see Figure 4).

**Figure 4:**
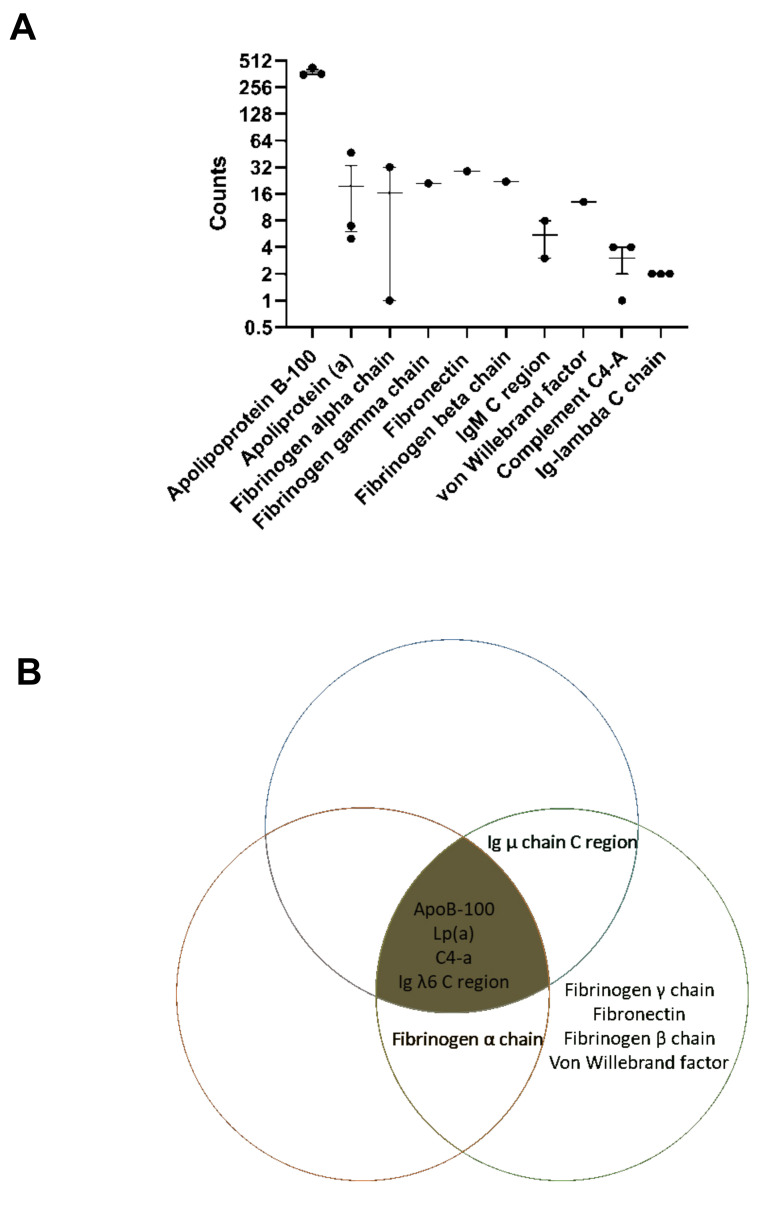
**LO1 immunoprecipitates ApoB-100.** Material was immunoprecipitated with LO1 and analysed by LC-MS/MS. (**A**) Number of counts of peptides from immunoprecipitated samples, with ApoB-100 counts twenty-fold higher than for any other protein peptide detected. Values bars are mean ± SEM, n = 3; (**B**) ApoB-100, Lp (a), C4-a and g λ6 C-region peptides were detected in LO1 immunoprecipitates of each of three plasma samples. Fibrinogen α chain and Ig μ chain C region were also detected in two patient samples. Each Venn circle represents one sample.

Not only can the capture ELISA measure MDA-LDL synthesized in the laboratory, but it also detects the presence of antigen in human plasma samples at varying concentration (Figure 3A,B). To establish that the LO1-reactive signal from plasma was indeed due to MDA-LDL, we first immunoprecipitated antigen from plasma with LO1 and then detected with Coomassie staining (Figure 3C(i)). A clear band was detected around 200 kD, which was significantly lower than that of laboratory generated MDA-LDL (above 210 kD), but slightly higher than native LDL (Figure 3C(ii)). Importantly, no band was obtained when the procedure was repeated with native unmodified LDL (not shown). The bands immunoprecipitated by LO1 from plasma of three patients were excised from the polyacrylamide gels, processed by trypsin digestion and reduction of cysteine residues with dithiothreitol and then further analyzed by LC-MS/MS. Maximal sequence coverage of ApoB-100 was 76%. ApoB-100 peptides were detected in all three patients at seven-fold higher levels than the next most abundant protein detected from the gel band (Figure 4A). Of the top 10 protein identifications (Supplementary Table S2), 2 were for bovine origin, likely originating from the experimental procedure with use of bovine albumin or trypsin. In addition to ApoB-100, LC-MS/MS of the LO1-immunoprecipitated material also identified proteins known to associate with oxLDL, including Apolipoprotein-a, IgM constant region and Complement C4a, but in lower abundance in total peptides identified when compared to ApoB-100 (Figure 4B).

**Figure 5:**
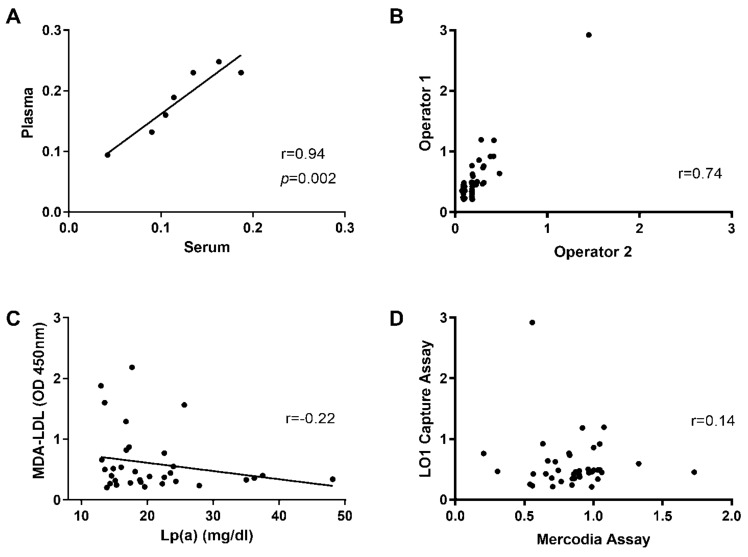
**Reproducibility of the LO1 capture assay.** (**A**) Correlation between the levels of MDA-LDL in plasma and serum in seven patients. Spearman r = 0.94; (**B**) Reproducibility shown for LO1 antibody capture assays by different operators, Spearman r = 0.74; (**C**) Correlation between MDA-LDL and Lipoprotein (a) (r = −0.22); (**D**) Comparison between LO1 antibody capture and Mercodia assay, showing no correlation between the assays (r = 0.14).

There was a strong positive correlation for MDA-LDL levels in paired plasma and serum samples (r = 0.94, *p* = 0.002, Figure 5A). High reproducibility for detecting MDA-LDL was achieved by different operators performing the assays at different times (r = 0.74, *p* < 0.0001, Figure 5B). Interestingly, no correlation was found with an assay detecting Lp (a) (R^2^ = 0.05, *p* =−0.22) (Figure 5C) or with a commercial assay using the 4E6 antibody (Mercodia, Uppsala, Sweden) (r = 0.14, Figure 5D).

Stress events, such as major surgery or overwhelming infections, result in a systemic inflammatory response, which can include free radical generation and upregulated proinflammatory signaling. We next sought to assess whether our MDA-LDL capture assay could detect dynamic changes in plasma samples following such an event, focusing on ten patients undergoing CABG surgery. Simultaneously, we also looked for dynamic changes in total ApoB, as well as IgM and IgG antibodies against MDA-LDL [15,16].

The patients were aged 67.1 (±9.0, standard deviation) years old and 2/10 were female. There was a high preponderance of cardiovascular risk factors, with 4/10 having had previous MI, 6/10 had diabetes mellitus, 5/10 were current or ex-smokers and 8/10 were hypertensive. Pre-operative left ventricular function was good in 9/10 and moderately impaired in 1/10. 8/10 had three-vessel coronary disease, whilst 2/10 had one or two-vessel disease. The cross-clamp time was 33.9 ± 6.9 min.

**Figure 6:**
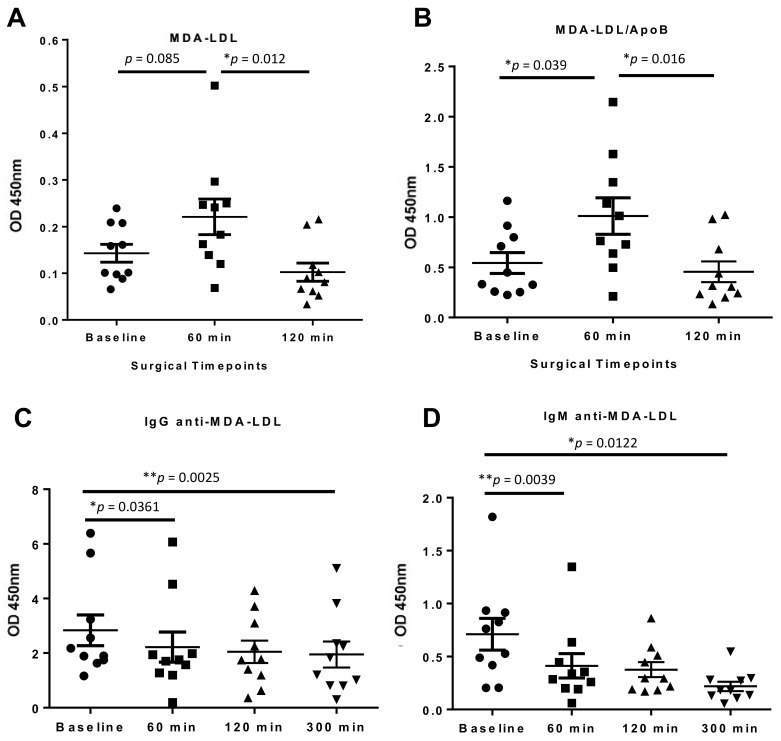
**LO1 detected temporal changes in MDA-LDL post CABG surgery (n = 10).** (**A**) MDA-LDL levels increased by 60 min post-operatively, returning to below baseline at 120 min; (**B**) Normalisation of MDA-LDL levels to ApoB demonstrates a significant increase in MDA-LDL levels 60 min post-CABG surgery; (**C**) IgG anti-MDA-LDL levels and (**D**) IgM anti-MDA-LDL levels decreased over 300 min post-CABG surgery. Values are mean ± SEM. * = *p* <0.05; ** = *p* <0.01.

Between baseline and 60 min postoperatively there was a non-significant increase in MDA-LDL (*p* = 0.085), measured using the LO1 sandwich ELISA assay, which then subsequently decreases at 120 min, to below baseline levels (*p* = 0.012 vs. 60 min) (Figure 6A). The same dynamic was seen when the MDA-LDL levels were normalized to ApoB to correct for possible dilutional and fluid changes, with the initial increase in MDA-LDL becoming statistically significant (*p* = 0.039) (Figure 6B). IgG anti-MDA-LDL antibody levels were reduced significantly from 60 min, and the reduction persisted to 300 min (*p* = 0.0361 and *p* = 0.0025 respectively) (Figure 6C). In contrast, there were no significant changes in total IgG levels at any timepoint, although a downward trend was noted (Supplementary Figure S1A). IgM anti-MDA-LDL antibody levels reduced significantly at 60 min (*p* = 0.0039) and 300 min (*p* = 0.0122) (Figure 6D). As for IgG, total IgM levels were unchanged between baseline and 60 min, although they did decline significantly at 300 min (Supplementary Figure S1B).

## 4. Discussion

This study has developed a novel assay for the convenient and robust measurement of MDA-LDL in fluid phase by ELISA; established that the assay can measure MDA-LDL in both human plasma and serum; and illustrated the applicability of the assay for detecting dynamic changes in plasma MDA-LDL in patients undergoing cardiopulmonary bypass during coronary artery bypass grafting. The assay is highly reproducible and can be used on samples frozen for twenty months (and possibly more) and showed no loss of sensitivity with samples subjected to multiple freeze-thaw cycles.

Unlike other assays which measure MDA in general, our assay using LO1 specifically measures fluid-phase MDA conjugated to LDL. There are three reasons for this assertion. First, our previous work had established that whilst LO1 recognizes MDA-LDL, it does not recognize MDA conjugated to albumin [12]. Secondly, material immunoprecipitated by LO1 from human plasma could be detected by Coomassie staining. The identified band ran slightly above that of unmodified LDL, but well below that of MDA-LDL prepared in the laboratory, suggesting that the assay is detecting LDL that is well below maximal MDA conjugation. We then excised the LO1-immunopreciptated bands >210 kD from an SDS-PAGE gel with three patient samples, and found by mass spectrometry in each of the patient samples that ApoB-100 was the predominant protein. Interestingly, other proteins identified in these samples by mass spectrometry, albeit with lower abundance based on detected peptides, included complement C4A and IgM. Assuming these are the residua of proteins complexed and co-immunoprecipitated with LDL, this is consistent with the postulated homeostatic role of the classical complement pathway and natural IgM antibodies in modified-LDL clearance [17,18,19].

Our interest in the homeostatic clearance of immune complexes based on modified LDL provides the background for the choice of cardiopulmonary bypass to test the applicability of the assay. Given the known ability of cardiopulmonary bypass to cause oxidative stress, we decided to use this to illustrate the utility of the assay by investigating dynamic changes in MDA-LDL levels. Once adjusted to ApoB levels to mitigate against dilutional effects of the procedure, MDA-LDL levels significantly rose at one hour from baseline but returned to preoperative levels by two hours. It is interesting to compare this result with those of a recent study which focused on derivatisable (i.e., free) MDA, as measured by a relatively elaborate gas chromatography-mass spectrometry method. In that study, free MDA rose with bypass and remained raised for several hours afterwards [20]. The drop over time that we observed in IgM and IgG anti-MDA-LDL antibodies is consistent with a consumptive phenomenon due to clearance of MDA-LDL immune complexes. Taken together, these data indicate that the level of MDA-LDL cannot be inferred from that of the overall pool of free MDA due to effective clearance mechanisms for MDA-conjugated proteins and attest to the value of specific assays such as that we have developed.

It is notable that the results from our assay showed a lack of correlation with Lp (a) levels, despite a high proportion of oxidized phospholipids associated with ApoB lipoproteins being found on Lp (a) [21]. Furthermore, the LO1 assay results failed to correlate with those from a commonly used commercial assay using the 4E6 antibody. The latter finding attests to the complexity of the oxidative modifications of LDL and the potential for greater understanding of the biochemistry in relation to clinical consequences through the development of a panel of complementary assays targeting distinct antigenic entities. Therefore, measuring MDA-LDL levels using our assay may provide further insights into both oxidative modification of lipoproteins dynamically, but also as a risk profile determinant as part of a matrix of biomarkers. This of course needs to be determined in other studies.

In particular, measurement of MDA-LDL, in comparison to other approaches, may provide important information relating to oxidative processing in response to stress events. We have demonstrated in this study that our assay is capable of sensitively detecting changes in MDA-LDL levels in coronary artery bypass grafting surgery, which would be directly translatable into other situations where the extent of oxidative stress on lipoproteins is unknown and needs to be determined. Furthermore, we anticipate that further risk profiling studies in large cohorts will be required to establish the value of this assay in predicting major adverse cardiovascular events in general populations. We hope that this novel work will provide a solid scientific basis for taking the assay into clinical translation at scale.

## 5. Conclusions

In conclusion, we have developed a novel capture ELISA for the measurement of MDA-LDL in body fluid. Further work in combination with existing assays may be instrumental in improving risk assessment of cardiovascular disease and other pathologies.

## Data Availability

Fully anonymised data presented in this study can potentially be available upon specific request at the discretion of the corresponding author. The data are not publicly available for privacy and ethical reasons given that the research participants who consented for the study did not provide specific consent to have their data shared in a public database.

## Supplementary Materials

The following are available online at https://www.mdpi.com/article/10.3390/antiox10081298/s1, Figure S1: Changes in total serum IgG and IgM post CABG surgery (n = 10), Table S1: Inclusion and exclusion criteria for the CABG surgery study, Table S2: Top 10 protein identifications from LC-MS/MS on three plasma samples.

## Abbreviations

ApoB	apolipoprotein B-100
BSA	bovine serum albumin
CABG	coronary artery bypass grafting
CVD	cardiovascular disease
ELISA	enzyme-linked immunosorbent assay
IgG	immunoglobulin G
IgM	immunoglobulin M
LDL	low-density lipoprotein
LDS	lithium dodecyl sulfate
Lp (a)	lipoprotein A
mAb	monoclonal antibody
MDA	malondialdehyde
MDA-LDL	malondialdehyde-conjugated LDL
OD	optical density
OSE	oxidation specific epitope
oxLDL	oxidized low-density lipoprotein
oxPL	oxidized phospholipids
PVDF	polyvinylidene fluoride
RT	room temperature
