# Supplementary material for: A Novel Immunoassay for Malondialdehyde-Conjugated Low-Density Lipoprotein Measures Dynamic Changes in the Blood of Patients Undergoing Coronary Artery Bypass Graft Surgery
Source: Antioxidants (Basel). 2021 Aug 17;10(8):1298. doi: 10.3390/antiox10081298 (PMC8389242; doi:10.3390/antiox10081298)
Supplement: Supplementary file 1 [file antioxidants-10-01298-s001.zip › antioxidants-1298384-supplementary.pdf]

antioxidants

Supplementary Materials: A Novel Immunoassay for Malondialdehyde-Conjugated Low-Density Lipoprotein Measures Dynamic Changes in the Blood of Patients Undergoing Coronary Artery Bypass Graft Surgery

Samata S. Pandey [1,†], Adam Hartley [1,†], Mikhail Caga-Anan [1], Tareq Ammari [1], Ameer Hamid Ahmed Khan [1], Bao Anh Vu Nguyen [1], Chiari Kojima [1], Jon Anderson [2], Steven Lynham [3], Michael Johns [1], Dorian O. Haskard [1] and Ramzi Y. Khamis [1,*]

[1] Vascular Sciences Section, National Heart and Lung Institute, Imperial Centre for Translational and Experimental Medicine, Imperial College London, London W12 0NN, UK; samata.pandey12@imperial.ac.uk (S.P.); adam.hartley12@imperial.ac.uk (A.H.); m-caga.anan@imperial.ac.uk (M.C.-A.); Tareq.ammari@gmail.com (T.A.); ameerhamidahmedkhan@gmail.com (A.H.A.K.); bao.nguyen@doctors.net.uk (B.A.V.N.); c.kojima@imperial.ac.uk (C.K.); m.johns@imperial.ac.uk (M.J.); d.haskard@imperial.ac.uk (D.O.H.)
[2] Department of Cardiothoracic Surgery, Hammersmith Hospital, Imperial College Healthcare NHS Trust, London, W12 0HS, UK; jonr.anderson@nhs.net
[3] Centre of Excellence for Mass Spectrometry, Proteomics Facility, Denmark Hill Campus, Kings College London, London, SE5 9NU, UK; steven.lynham@kcl.ac.uk
† Equally contributed: samata.pandey12@imperial.ac.uk (S.P.); adam.hartley12@imperial.ac.uk (A.H.)
* Corresponding author: ramzi.khamis@imperial.ac.uk; Tel.: +44-(020)-7594-6842

Citation: Pandey, S.S.; Hartley, A.; Caga-Anan, M.; Ammari, T.; Khan, A.H.A.; Nguyen, B.A.V.; Kojima, C.; Anderson, J.; Lynham, S.; Johns, M.; et al. A Novel Immunoassay for Malondialdehyde-Conjugated Low-Density Lipoprotein Measures Dynamic Changes in the Blood of Patients Undergoing Coronary Artery Bypass Graft Surgery. *Antioxidants* **2021**, *10*, 1298. https://doi.org/10.3390/antiox10081298

Academic Editor: Fumiaki Ito

Received: 29 June 2021
Accepted: 10 August 2021
Published: 17 August 2021

Publisher's Note: MDPI stays neutral with regard to jurisdictional claims in published maps and institutional affiliations.

Copyright: © 2021 by the authors. Licensee MDPI, Basel, Switzerland. This article is an open access article distributed under the terms and conditions of the Creative Commons Attribution (CC BY) license (http://creativecommons.org/licenses/by/4.0/).

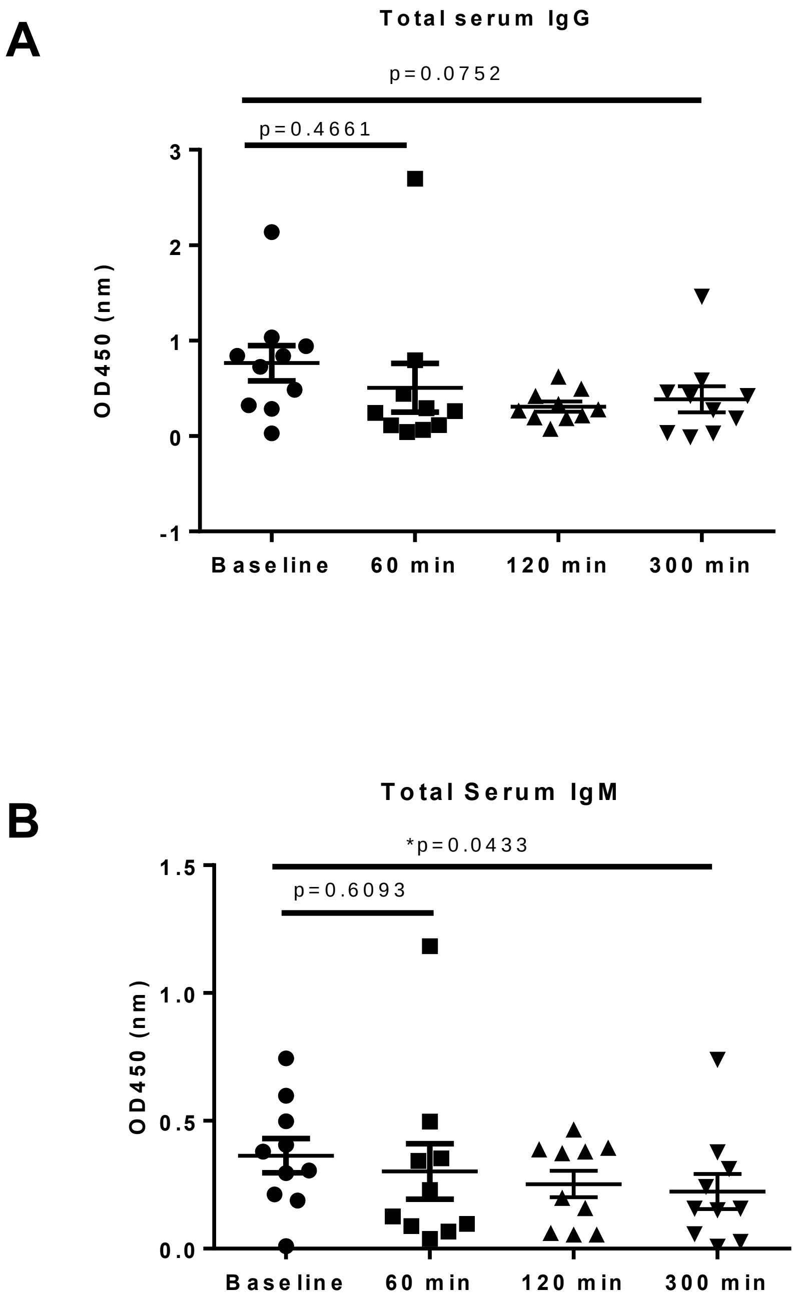

Figure S1. Changes in total serum IgG and IgM post CABG surgery (n=10). A) Total IgG did not change from baseline to 300 minutes B) IgM decreased from baseline to 300 minutes. Values are mean ± SEM.

TableS1. Inclusion and exclusion criteria for the patients undergoing coronary artery bypass grafting in the population study. CABG, coronary artery bypass grafting.

Inclusion Criteria	Patients referred for primary elective CABG surgery at Hammersmith Hospital were considered for inclusion
Exclusion Criteria	-Paediatric patients<br>-Emergency surgeries (within 24 hours)<br>-Combined CABG and valve replacement<br>-Re-do operations with re-sternotomy

-Poor left ventricular function (ejection fraction <30% on echocardiogram)
-Recent cerebrovascular accident within 3 months pre-operatively/> 75% carotid artery obstruction on ultrasound
-Pre-existing renal impairment (serum creatinine>177μmol/L)
-Pre-existing coagulopathy
-Pre-existing liver dysfunction
-Recent (within 5 days) use of antiplatelet agents (aspirin,clopidrogel)

Table S2. The top 10 protein identifications from each patient using LC-MS/MS. ApoB-100 peptides were detected in all three patients at seven-fold higher levels than the next most abundant protein. Unless stated otherwise, origin of protein was identified as Homo sapiens.

**Patient 1**		**Patient 2**		**Patient 3**	
Proteins identified	*Counts*	*Proteins identified*	*Counts*	*Proteins identified*	*Counts*
Apolipoprotein B-100	363	Apolipoprotein B-100	429	Apolipoprotein B-100	354
Cationic trypsin (Bos taurus)	14	Apolipoprotein(a)	47	Cationic trypsin (Bos taurus)	13
Apolipoprotein(a)	7	Fibrinogen alpha chain	32	Apolipoprotein(a)	5
Complement C4-A	4	Fibronectin	29	Serum albumin (Bos taurus)	5
Ig mu chain C region	3	Fibrinogen beta chain	22	Ig lambda-6 chain C region	2
Serum albumin (Bos taurus)	2	Fibrinogen gamma chain	21	Fibrinogen alpha chain	1
Ig lambda-6 chain C region	2	von Willebrand factor	13	Complement C4-A	1
Anionic trypsin (Rattus norvegicus)	1	Serum albumin (Bos taurus)	11	Anionic trypsin (Rattus norvegicus)	1
Ig gamma 4 chain C region	1	Cationic trypsin (Bos taurus)	10	Ig gamma 4 chain C region	1
Ig lambda chain V region	1	Ig mu chain C region	8	Hornerin	1
